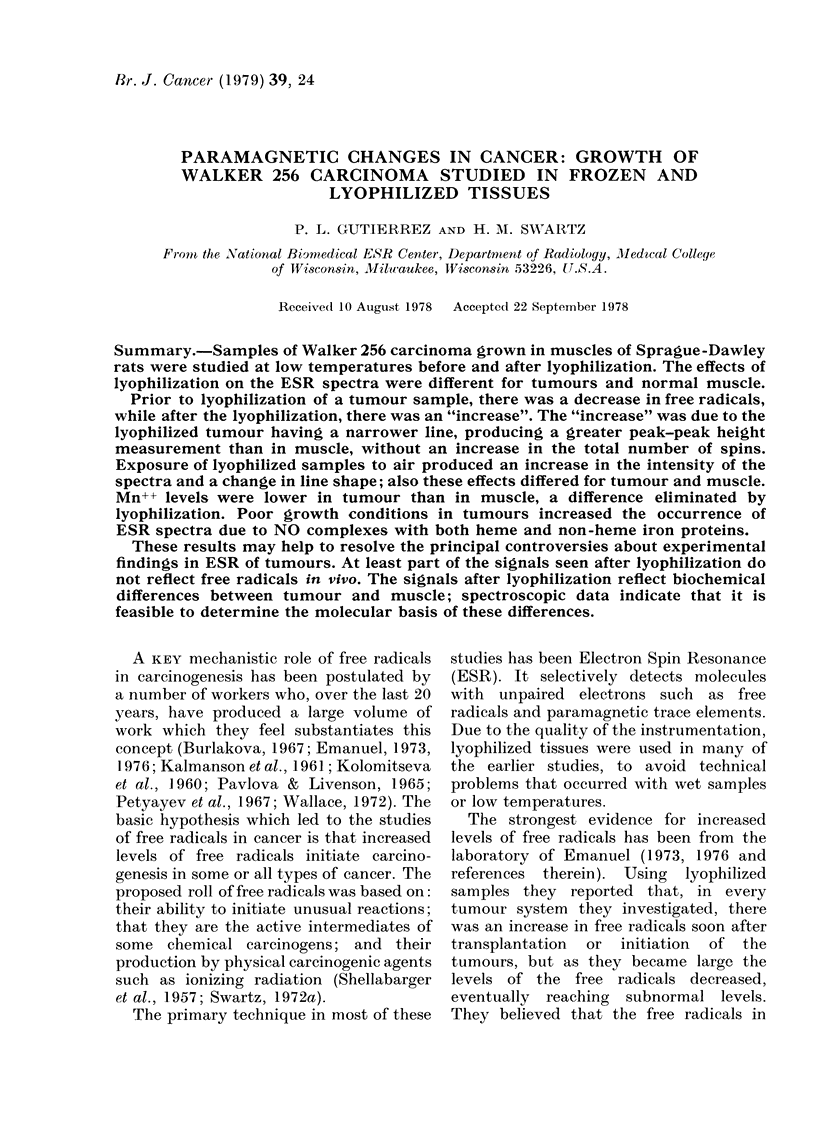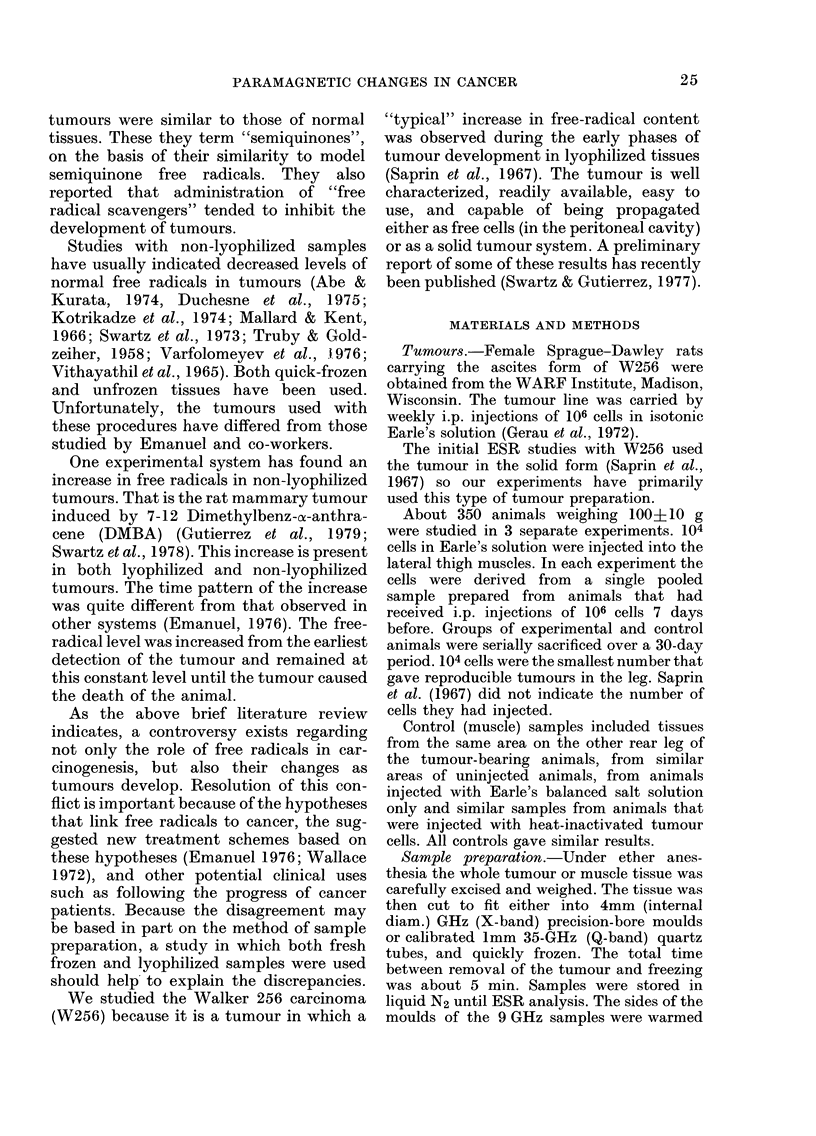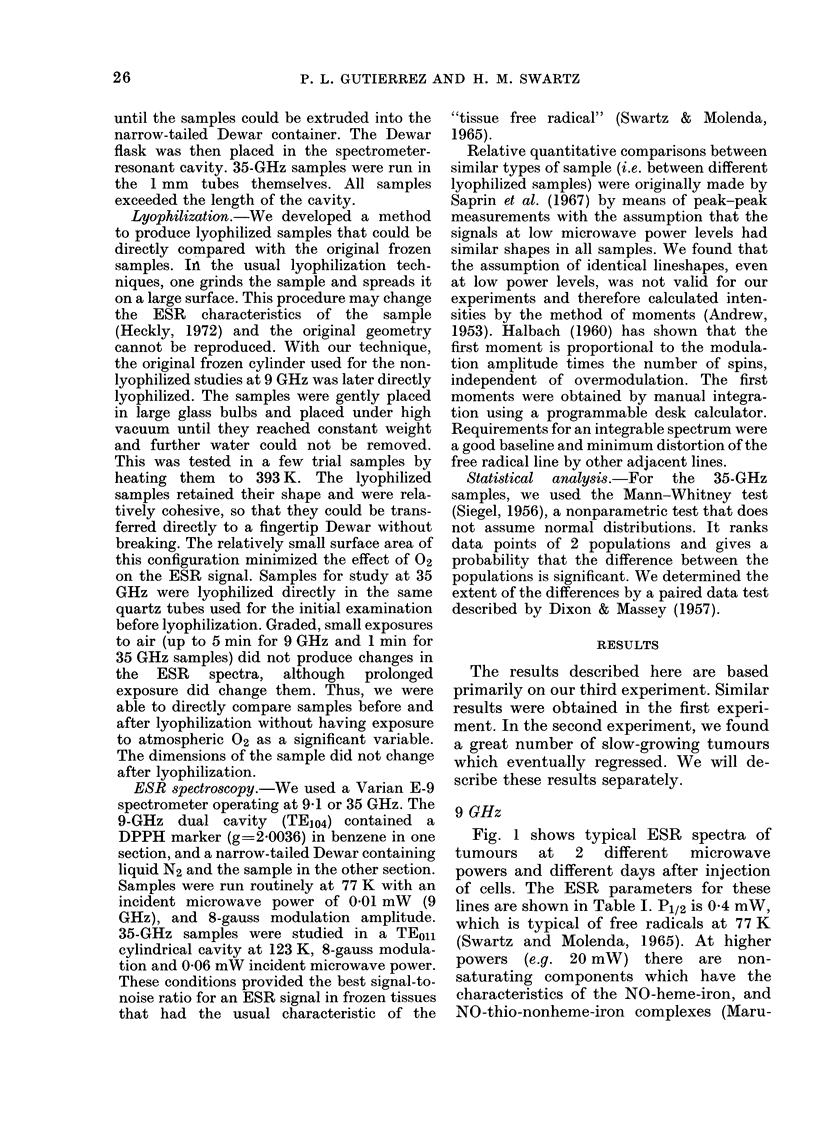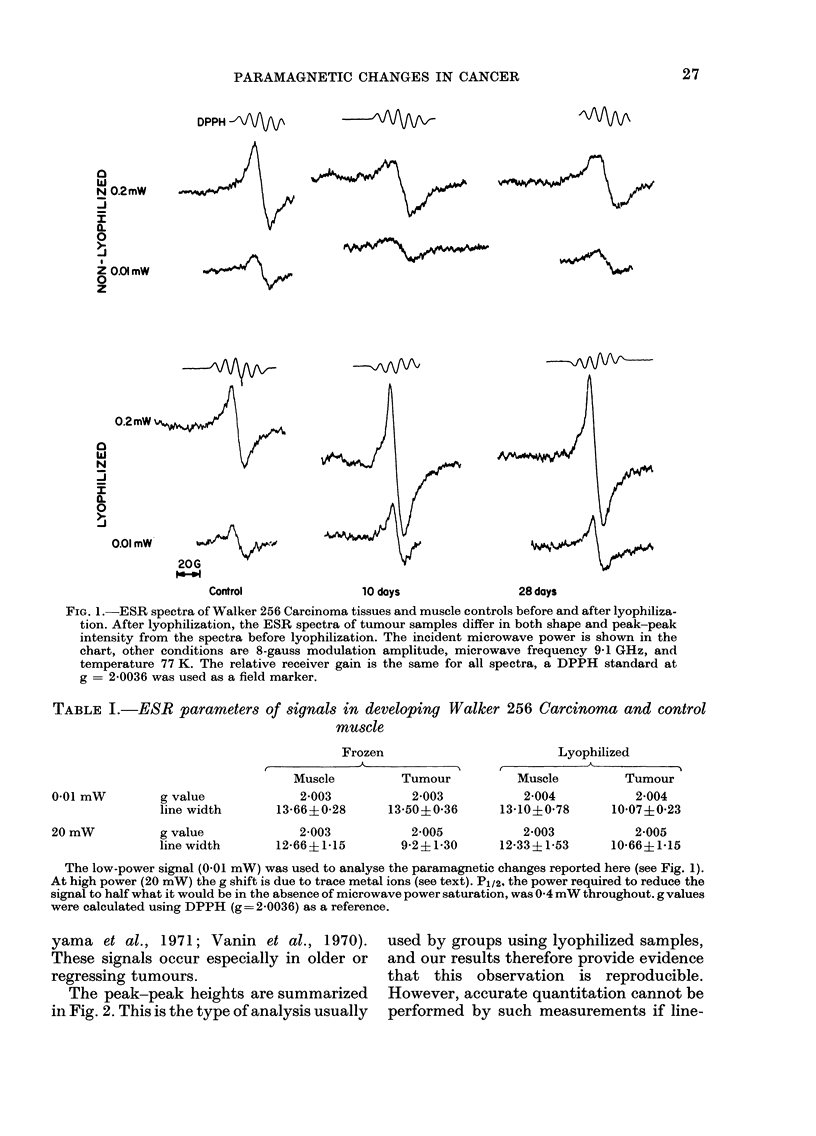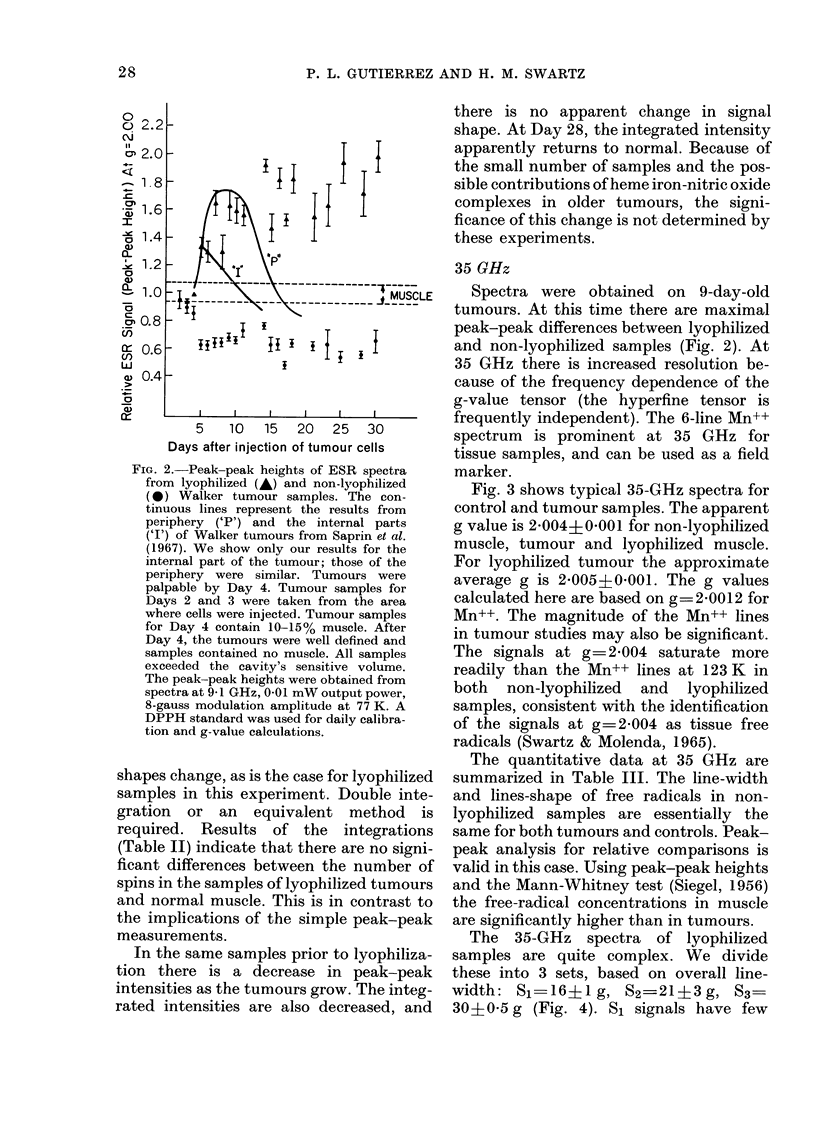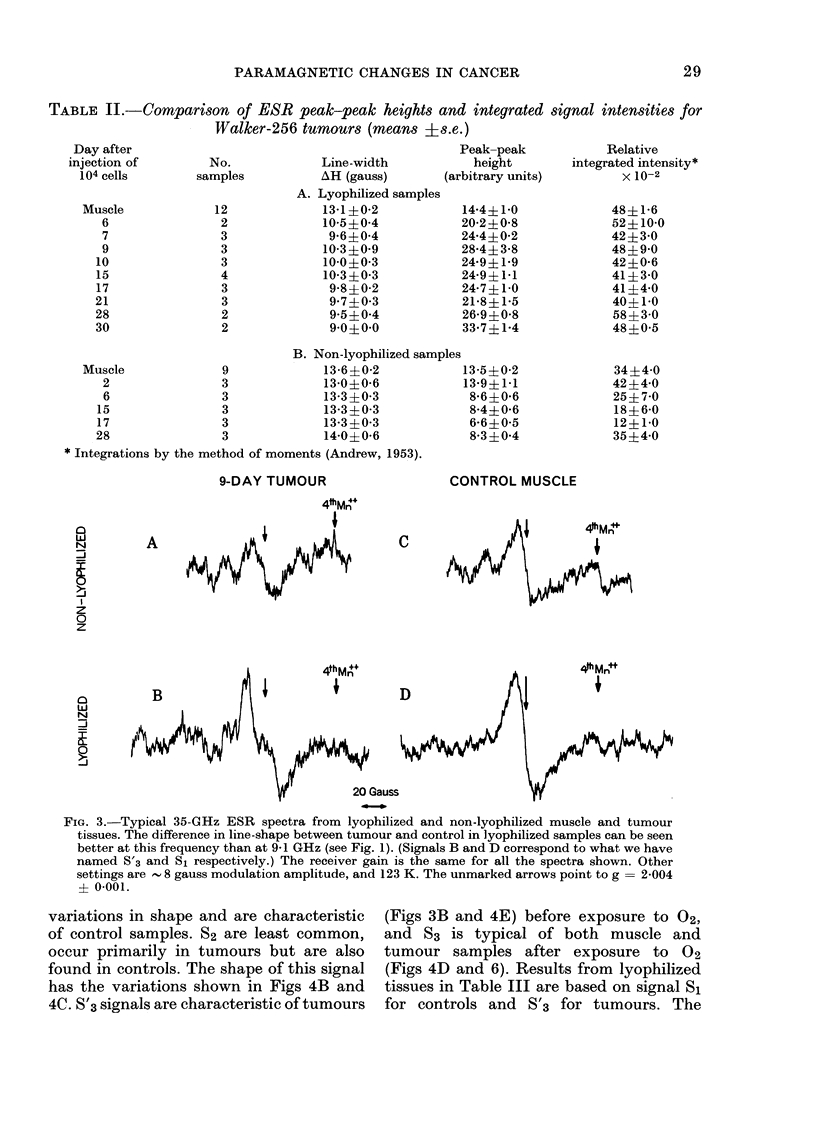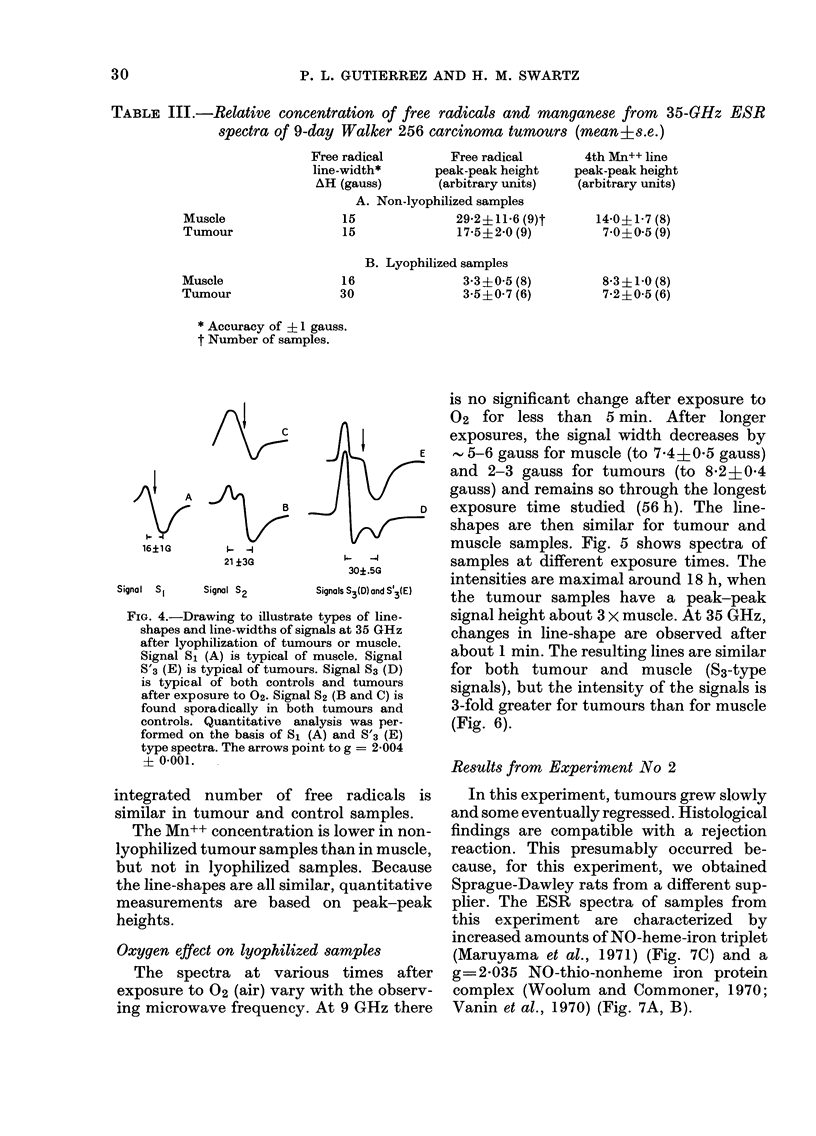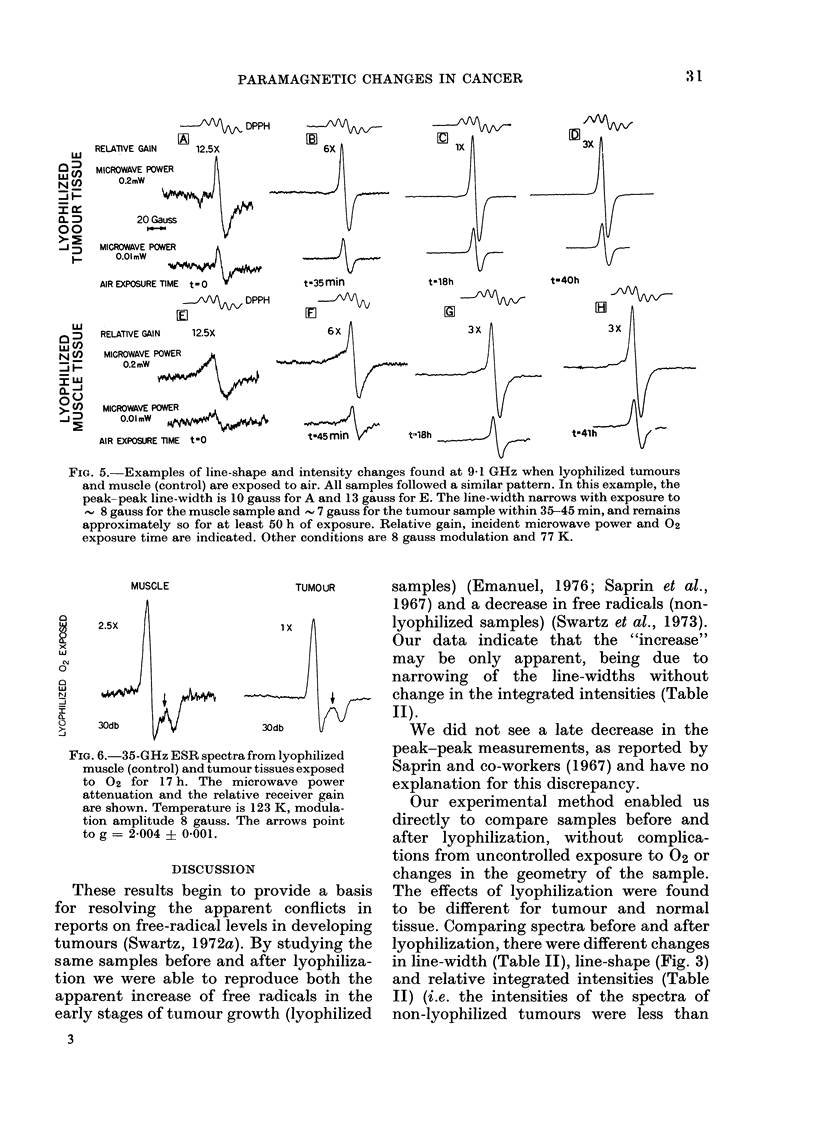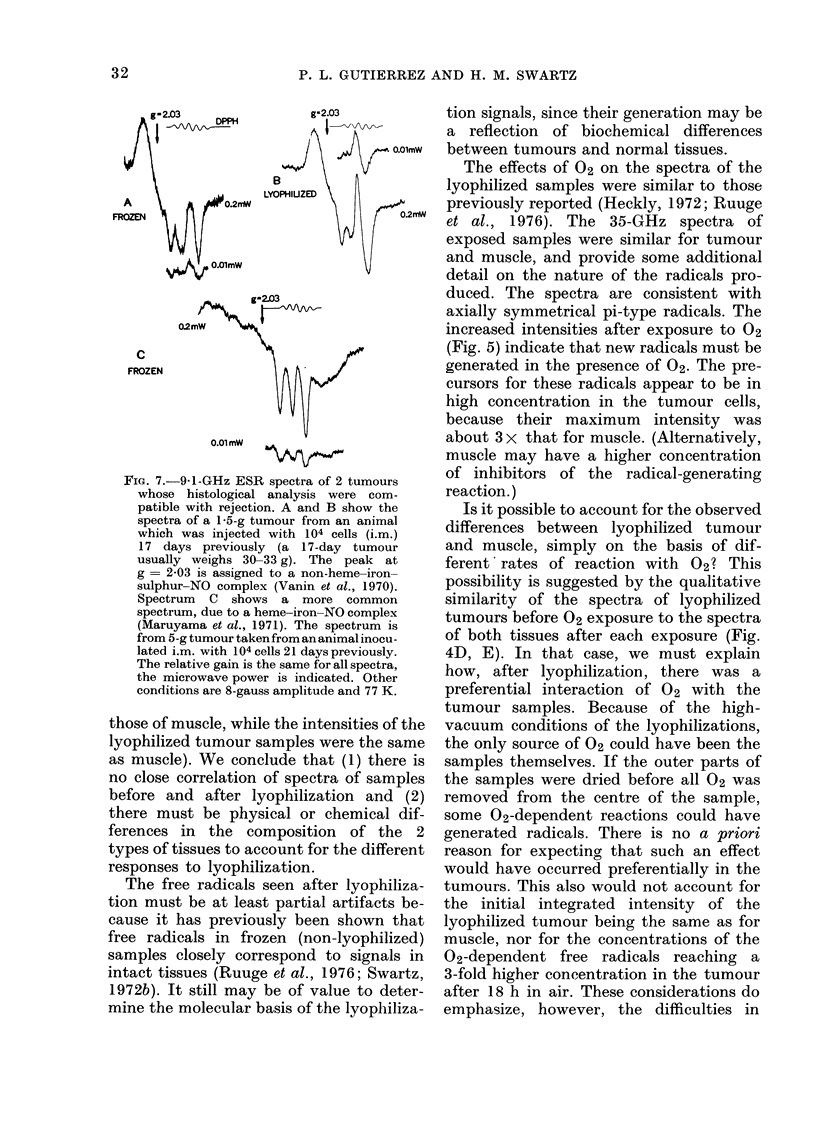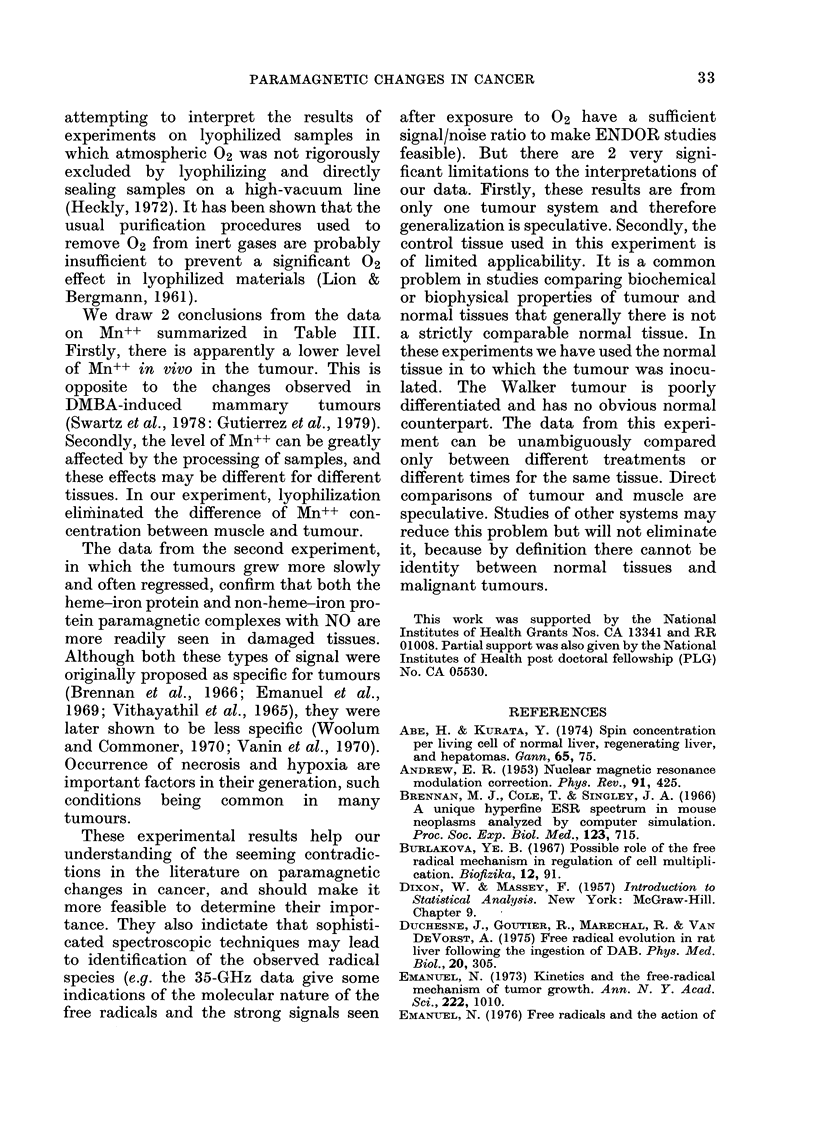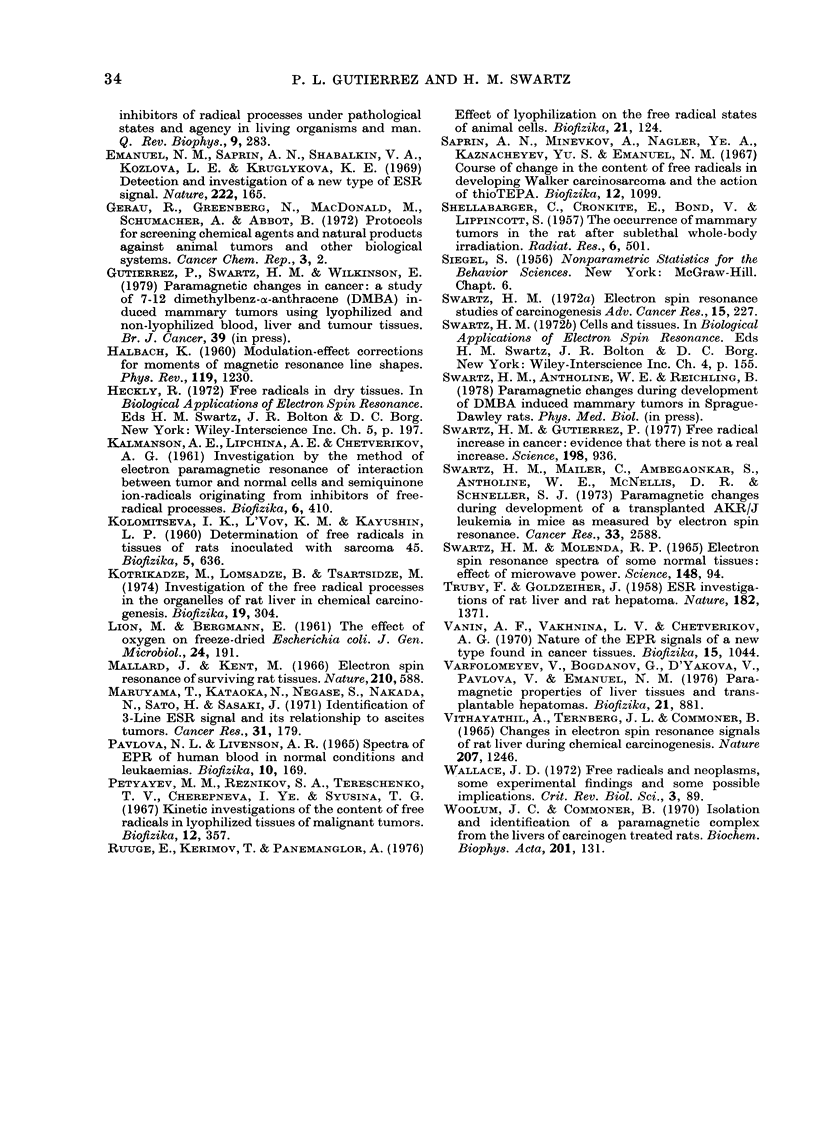# Paramagnetic changes in cancer: growth of Walker 256 carcinoma studied in frozen and lyophilized tissues.

**DOI:** 10.1038/bjc.1979.4

**Published:** 1979-01

**Authors:** P. L. Gutierrez, H. M. Swartz

## Abstract

Samples of Walker 256 carcinoma grown in muscles of Sprague-Dawley rats were studied at low temperatures before and after lyophilization. The effects of lyophilization on the ESR spectra were different for tumours and normal muscle. Prior to lyophilization of a tumour sample, there was a decrease in free radicals, while after the lyophilization, there was an "increase". The "increase" was due to the lyophilized tumour having a narrower line, producing a greater peak-peak height measurement than in muscle, without an increase in the total number of spins. Exposure of lyophilized samples to air produced an increase in the intensity of the spectra and a change in line shape; also these effects differed for tumour and muscle. Mn++ levels were lower in tumour than in muscle, a difference eliminated by lyophilization. Poor growth conditions in tumours increased the occurrence of ESR spectra due to NO complexes with both heme and non-heme iron proteins. These results may help to resolve the principal controversies about experimental findings in ESR of tumours. At least part of the signals seen after lyophilization do not reflect free radicals in vivo. The signals after lyophilization reflect biochemical differences between tumour and muscle; spectroscopic data indicate that it is feasible to determine the molecular basis of these differences.


Br. J. Cancer (1979) 39, 24

### PARAMAGNETIC CHANGES IN CANCER: GROWTH OF WALKER 256 CARCINOMA STUDIED IN FROZEN AND LYOPHILIZED TISSUES

P. L. GUTIERREZ AND H. M. SWARTZ

*From the National Biomedical ESR Center, Department of Radiology, Medical College of Wisconsin, Milwaukee, Wisconsin 53226, U.S.A.*

Received 10 August 1978 Accepted 22 September 1978

**Summary.—Samples of Walker 256 carcinoma grown in muscles of Sprague-Dawley rats were studied at low temperatures before and after lyophilization. The effects of lyophilization on the ESR spectra were different for tumours and normal muscle.**

**Prior to lyophilization of a tumour sample, there was a decrease in free radicals, while after the lyophilization, there was an "increase". The "increase" was due to the lyophilized tumour having a narrower line, producing a greater peak–peak height measurement than in muscle, without an increase in the total number of spins. Exposure of lyophilized samples to air produced an increase in the intensity of the spectra and a change in line shape; also these effects differed for tumour and muscle. $Mn^{++}$ levels were lower in tumour than in muscle, a difference eliminated by lyophilization. Poor growth conditions in tumours increased the occurrence of ESR spectra due to NO complexes with both heme and non-heme iron proteins.**

**These results may help to resolve the principal controversies about experimental findings in ESR of tumours. At least part of the signals seen after lyophilization do not reflect free radicals *in vivo*. The signals after lyophilization reflect biochemical differences between tumour and muscle; spectroscopic data indicate that it is feasible to determine the molecular basis of these differences.**

A KEY mechanistic role of free radicals in carcinogenesis has been postulated by a number of workers who, over the last 20 years, have produced a large volume of work which they feel substantiates this concept (Burlakova, 1967; Emanuel, 1973, 1976; Kalmanson *et al.*, 1961; Kolomitseva *et al.*, 1960; Pavlova & Livenson, 1965; Petyayev *et al.*, 1967; Wallace, 1972). The basic hypothesis which led to the studies of free radicals in cancer is that increased levels of free radicals initiate carcinogenesis in some or all types of cancer. The proposed roll of free radicals was based on: their ability to initiate unusual reactions; that they are the active intermediates of some chemical carcinogens; and their production by physical carcinogenic agents such as ionizing radiation (Shellabarger *et al.*, 1957; Swartz, 1972*a*).

The primary technique in most of these studies has been Electron Spin Resonance (ESR). It selectively detects molecules with unpaired electrons such as free radicals and paramagnetic trace elements. Due to the quality of the instrumentation, lyophilized tissues were used in many of the earlier studies, to avoid technical problems that occurred with wet samples or low temperatures.

The strongest evidence for increased levels of free radicals has been from the laboratory of Emanuel (1973, 1976 and references therein). Using lyophilized samples they reported that, in every tumour system they investigated, there was an increase in free radicals soon after transplantation or initiation of the tumours, but as they became large the levels of the free radicals decreased, eventually reaching subnormal levels. They believed that the free radicals in tumours were similar to those of normal tissues. These they term "semiquinones", on the basis of their similarity to model semiquinone free radicals. They also reported that administration of "free radical scavengers" tended to inhibit the development of tumours.

Studies with non-lyophilized samples have usually indicated decreased levels of normal free radicals in tumours (Abe & Kurata, 1974, Duchesne *et al.*, 1975; Kotrikadze *et al.*, 1974; Mallard & Kent, 1966; Swartz *et al.*, 1973; Truby & Goldzeiher, 1958; Varfolomeyev *et al.*, 1976; Vithayathil *et al.*, 1965). Both quick-frozen and unfrozen tissues have been used. Unfortunately, the tumours used with these procedures have differed from those studied by Emanuel and co-workers.

One experimental system has found an increase in free radicals in non-lyophilized tumours. That is the rat mammary tumour induced by 7-12 Dimethylbenz-$\alpha$-anthracene (DMBA) (Gutierrez *et al.*, 1979; Swartz *et al.*, 1978). This increase is present in both lyophilized and non-lyophilized tumours. The time pattern of the increase was quite different from that observed in other systems (Emanuel, 1976). The free-radical level was increased from the earliest detection of the tumour and remained at this constant level until the tumour caused the death of the animal.

As the above brief literature review indicates, a controversy exists regarding not only the role of free radicals in carcinogenesis, but also their changes as tumours develop. Resolution of this conflict is important because of the hypotheses that link free radicals to cancer, the suggested new treatment schemes based on these hypotheses (Emanuel 1976; Wallace 1972), and other potential clinical uses such as following the progress of cancer patients. Because the disagreement may be based in part on the method of sample preparation, a study in which both fresh frozen and lyophilized samples were used should help to explain the discrepancies.

We studied the Walker 256 carcinoma (W256) because it is a tumour in which a "typical" increase in free-radical content was observed during the early phases of tumour development in lyophilized tissues (Saprin *et al.*, 1967). The tumour is well characterized, readily available, easy to use, and capable of being propagated either as free cells (in the peritoneal cavity) or as a solid tumour system. A preliminary report of some of these results has recently been published (Swartz & Gutierrez, 1977).

### MATERIALS AND METHODS

*Tumours.*—Female Sprague–Dawley rats carrying the ascites form of W256 were obtained from the WARF Institute, Madison, Wisconsin. The tumour line was carried by weekly i.p. injections of $10^6$ cells in isotonic Earle's solution (Gerau *et al.*, 1972).

The initial ESR studies with W256 used the tumour in the solid form (Saprin *et al.*, 1967) so our experiments have primarily used this type of tumour preparation.

About 350 animals weighing $100 \pm 10$ g were studied in 3 separate experiments. $10^4$ cells in Earle's solution were injected into the lateral thigh muscles. In each experiment the cells were derived from a single pooled sample prepared from animals that had received i.p. injections of $10^6$ cells 7 days before. Groups of experimental and control animals were serially sacrificed over a 30-day period. $10^4$ cells were the smallest number that gave reproducible tumours in the leg. Saprin *et al.* (1967) did not indicate the number of cells they had injected.

Control (muscle) samples included tissues from the same area on the other rear leg of the tumour-bearing animals, from similar areas of uninjected animals, from animals injected with Earle's balanced salt solution only and similar samples from animals that were injected with heat-inactivated tumour cells. All controls gave similar results.

*Sample preparation.*—Under ether anesthesia the whole tumour or muscle tissue was carefully excised and weighed. The tissue was then cut to fit either into 4mm (internal diam.) GHz (X-band) precision-bore moulds or calibrated 1mm 35-GHz (Q-band) quartz tubes, and quickly frozen. The total time between removal of the tumour and freezing was about 5 min. Samples were stored in liquid $N_2$ until ESR analysis. The sides of the moulds of the 9 GHz samples were warmed until the samples could be extruded into the narrow-tailed Dewar container. The Dewar flask was then placed in the spectrometer-resonant cavity. 35-GHz samples were run in the 1 mm tubes themselves. All samples exceeded the length of the cavity.

*Lyophilization.*—We developed a method to produce lyophilized samples that could be directly compared with the original frozen samples. In the usual lyophilization techniques, one grinds the sample and spreads it on a large surface. This procedure may change the ESR characteristics of the sample (Heckly, 1972) and the original geometry cannot be reproduced. With our technique, the original frozen cylinder used for the non-lyophilized studies at 9 GHz was later directly lyophilized. The samples were gently placed in large glass bulbs and placed under high vacuum until they reached constant weight and further water could not be removed. This was tested in a few trial samples by heating them to 393 K. The lyophilized samples retained their shape and were relatively cohesive, so that they could be transferred directly to a fingertip Dewar without breaking. The relatively small surface area of this configuration minimized the effect of $O_2$ on the ESR signal. Samples for study at 35 GHz were lyophilized directly in the same quartz tubes used for the initial examination before lyophilization. Graded, small exposures to air (up to 5 min for 9 GHz and 1 min for 35 GHz samples) did not produce changes in the ESR spectra, although prolonged exposure did change them. Thus, we were able to directly compare samples before and after lyophilization without having exposure to atmospheric $O_2$ as a significant variable. The dimensions of the sample did not change after lyophilization.

*ESR spectroscopy.*—We used a Varian E-9 spectrometer operating at 9·1 or 35 GHz. The 9-GHz dual cavity ($TE_{104}$) contained a DPPH marker (g=2·0036) in benzene in one section, and a narrow-tailed Dewar containing liquid $N_2$ and the sample in the other section. Samples were run routinely at 77 K with an incident microwave power of 0·01 mW (9 GHz), and 8-gauss modulation amplitude. 35-GHz samples were studied in a $TE_{011}$ cylindrical cavity at 123 K, 8-gauss modulation and 0·06 mW incident microwave power. These conditions provided the best signal-to-noise ratio for an ESR signal in frozen tissues that had the usual characteristic of the "tissue free radical" (Swartz & Molenda, 1965).

Relative quantitative comparisons between similar types of sample (*i.e.* between different lyophilized samples) were originally made by Saprin *et al.* (1967) by means of peak–peak measurements with the assumption that the signals at low microwave power levels had similar shapes in all samples. We found that the assumption of identical lineshapes, even at low power levels, was not valid for our experiments and therefore calculated intensities by the method of moments (Andrew, 1953). Halbach (1960) has shown that the first moment is proportional to the modulation amplitude times the number of spins, independent of overmodulation. The first moments were obtained by manual integration using a programmable desk calculator. Requirements for an integrable spectrum were a good baseline and minimum distortion of the free radical line by other adjacent lines.

*Statistical analysis.*—For the 35-GHz samples, we used the Mann–Whitney test (Siegel, 1956), a nonparametric test that does not assume normal distributions. It ranks data points of 2 populations and gives a probability that the difference between the populations is significant. We determined the extent of the differences by a paired data test described by Dixon & Massey (1957).

### RESULTS

The results described here are based primarily on our third experiment. Similar results were obtained in the first experiment. In the second experiment, we found a great number of slow-growing tumours which eventually regressed. We will describe these results separately.

### 9 *GHz*

Fig. 1 shows typical ESR spectra of tumours at 2 different microwave powers and different days after injection of cells. The ESR parameters for these lines are shown in Table I. $P_{1/2}$ is 0·4 mW, which is typical of free radicals at 77 K (Swartz and Molenda, 1965). At higher powers (*e.g.* 20 mW) there are non-saturating components which have the characteristics of the NO-heme-iron, and NO-thio-nonheme-iron complexes (Maru-

FIG. 1.—ESR spectra of Walker 256 Carcinoma tissues and muscle controls before and after lyophilization. After lyophilization, the ESR spectra of tumour samples differ in both shape and peak–peak intensity from the spectra before lyophilization. The incident microwave power is shown in the chart, other conditions are 8-gauss modulation amplitude, microwave frequency 9·1 GHz, and temperature 77 K. The relative receiver gain is the same for all spectra, a DPPH standard at g = 2·0036 was used as a field marker.

TABLE I.—*ESR parameters of signals in developing Walker 256 Carcinoma and control muscle*

| | | Frozen | | Lyophilized | |
|---|---|---|---|---|---|
| | | Muscle | Tumour | Muscle | Tumour |
| 0·01 mW | g value | 2·003 | 2·003 | 2·004 | 2·004 |
| | line width | 13·66±0·28 | 13·50±0·36 | 13·10±0·78 | 10·07±0·23 |
| 20 mW | g value | 2·003 | 2·005 | 2·003 | 2·005 |
| | line width | 12·66±1·15 | 9·2±1·30 | 12·33±1·53 | 10·66±1·15 |

The low-power signal (0·01 mW) was used to analyse the paramagnetic changes reported here (see Fig. 1). At high power (20 mW) the g shift is due to trace metal ions (see text). $P_{1/2}$, the power required to reduce the signal to half what it would be in the absence of microwave power saturation, was 0·4 mW throughout. g values were calculated using DPPH (g = 2·0036) as a reference.

yama *et al.*, 1971; Vanin *et al.*, 1970). These signals occur especially in older or regressing tumours.

The peak–peak heights are summarized in Fig. 2. This is the type of analysis usually used by groups using lyophilized samples, and our results therefore provide evidence that this observation is reproducible. However, accurate quantitation cannot be performed by such measurements if line-

FIG. 2.—Peak-peak heights of ESR spectra from lyophilized (▲) and non-lyophilized (●) Walker tumour samples. The continuous lines represent the results from periphery ('P') and the internal parts ('I') of Walker tumours from Saprin *et al.* (1967). We show only our results for the internal part of the tumour; those of the periphery were similar. Tumours were palpable by Day 4. Tumour samples for Days 2 and 3 were taken from the area where cells were injected. Tumour samples for Day 4 contain 10–15% muscle. After Day 4, the tumours were well defined and samples contained no muscle. All samples exceeded the cavity's sensitive volume. The peak–peak heights were obtained from spectra at 9·1 GHz, 0·01 mW output power, 8-gauss modulation amplitude at 77 K. A DPPH standard was used for daily calibration and g-value calculations.

shapes change, as is the case for lyophilized samples in this experiment. Double integration or an equivalent method is required. Results of the integrations (Table II) indicate that there are no significant differences between the number of spins in the samples of lyophilized tumours and normal muscle. This is in contrast to the implications of the simple peak–peak measurements.

In the same samples prior to lyophilization there is a decrease in peak–peak intensities as the tumours grow. The integrated intensities are also decreased, and there is no apparent change in signal shape. At Day 28, the integrated intensity apparently returns to normal. Because of the small number of samples and the possible contributions of heme iron-nitric oxide complexes in older tumours, the significance of this change is not determined by these experiments.

### 35 *GHz*

Spectra were obtained on 9-day-old tumours. At this time there are maximal peak–peak differences between lyophilized and non-lyophilized samples (Fig. 2). At 35 GHz there is increased resolution because of the frequency dependence of the g-value tensor (the hyperfine tensor is frequently independent). The 6-line $Mn^{++}$ spectrum is prominent at 35 GHz for tissue samples, and can be used as a field marker.

Fig. 3 shows typical 35-GHz spectra for control and tumour samples. The apparent g value is $2{\cdot}004 \pm 0{\cdot}001$ for non-lyophilized muscle, tumour and lyophilized muscle. For lyophilized tumour the approximate average g is $2{\cdot}005 \pm 0{\cdot}001$. The g values calculated here are based on $g = 2{\cdot}0012$ for $Mn^{++}$. The magnitude of the $Mn^{++}$ lines in tumour studies may also be significant. The signals at $g = 2{\cdot}004$ saturate more readily than the $Mn^{++}$ lines at 123 K in both non-lyophilized and lyophilized samples, consistent with the identification of the signals at $g = 2{\cdot}004$ as tissue free radicals (Swartz & Molenda, 1965).

The quantitative data at 35 GHz are summarized in Table III. The line-width and lines-shape of free radicals in non-lyophilized samples are essentially the same for both tumours and controls. Peak–peak analysis for relative comparisons is valid in this case. Using peak–peak heights and the Mann-Whitney test (Siegel, 1956) the free-radical concentrations in muscle are significantly higher than in tumours.

The 35-GHz spectra of lyophilized samples are quite complex. We divide these into 3 sets, based on overall line-width: $S_1 = 16 \pm 1$ g, $S_2 = 21 \pm 3$ g, $S_3 = 30 \pm 0{\cdot}5$ g (Fig. 4). $S_1$ signals have few

TABLE II.—*Comparison of ESR peak–peak heights and integrated signal intensities for Walker-256 tumours (means ±s.e.)*

| Day after injection of $10^4$ cells | No. samples | Line-width ΔH (gauss) | Peak–peak height (arbitrary units) | Relative integrated intensity* $\times 10^{-2}$ |
|---|---|---|---|---|
| | | A. Lyophilized samples | | |
| Muscle | 12 | 13·1±0·2 | 14·4±1·0 | 48±1·6 |
| 6 | 2 | 10·5±0·4 | 20·2±0·8 | 52±10·0 |
| 7 | 3 | 9·6±0·4 | 24·4±0·2 | 42±3·0 |
| 9 | 3 | 10·3±0·9 | 28·4±3·8 | 48±9·0 |
| 10 | 3 | 10·0±0·3 | 24·9±1·9 | 42±0·6 |
| 15 | 4 | 10·3±0·3 | 24·9±1·1 | 41±3·0 |
| 17 | 3 | 9·8±0·2 | 24·7±1·0 | 41±4·0 |
| 21 | 3 | 9·7±0·3 | 21·8±1·5 | 40±1·0 |
| 28 | 2 | 9·5±0·4 | 26·9±0·8 | 58±3·0 |
| 30 | 2 | 9·0±0·0 | 33·7±1·4 | 48±0·5 |
| | | B. Non-lyophilized samples | | |
| Muscle | 9 | 13·6±0·2 | 13·5±0·2 | 34±4·0 |
| 2 | 3 | 13·0±0·6 | 13·9±1·1 | 42±4·0 |
| 6 | 3 | 13·3±0·3 | 8·6±0·6 | 25±7·0 |
| 15 | 3 | 13·3±0·3 | 8·4±0·6 | 18±6·0 |
| 17 | 3 | 13·3±0·3 | 6·6±0·5 | 12±1·0 |
| 28 | 3 | 14·0±0·6 | 8·3±0·4 | 35±4·0 |

* Integrations by the method of moments (Andrew, 1953).

FIG. 3.—Typical 35-GHz ESR spectra from lyophilized and non-lyophilized muscle and tumour tissues. The difference in line-shape between tumour and control in lyophilized samples can be seen better at this frequency than at 9·1 GHz (see Fig. 1). (Signals B and D correspond to what we have named $S'_3$ and $S_1$ respectively.) The receiver gain is the same for all the spectra shown. Other settings are ~8 gauss modulation amplitude, and 123 K. The unmarked arrows point to g = 2·004 ± 0·001.

variations in shape and are characteristic of control samples. $S_2$ are least common, occur primarily in tumours but are also found in controls. The shape of this signal has the variations shown in Figs 4B and 4C. $S'_3$ signals are characteristic of tumours (Figs 3B and 4E) before exposure to $O_2$, and $S_3$ is typical of both muscle and tumour samples after exposure to $O_2$ (Figs 4D and 6). Results from lyophilized tissues in Table III are based on signal $S_1$ for controls and $S'_3$ for tumours. The

TABLE III.—*Relative concentration of free radicals and manganese from 35-GHz ESR spectra of 9-day Walker 256 carcinoma tumours (mean$\pm$s.e.)*

| | Free radical line-width* ΔH (gauss) | Free radical peak-peak height (arbitrary units) | 4th $Mn^{++}$ line peak-peak height (arbitrary units) |
|---|---|---|---|
| | A. Non-lyophilized samples | | |
| Muscle | 15 | 29·2$\pm$11·6 (9)† | 14·0$\pm$1·7 (8) |
| Tumour | 15 | 17·5$\pm$2·0 (9) | 7·0$\pm$0·5 (9) |
| | B. Lyophilized samples | | |
| Muscle | 16 | 3·3$\pm$0·5 (8) | 8·3$\pm$1·0 (8) |
| Tumour | 30 | 3·5$\pm$0·7 (6) | 7·2$\pm$0·5 (6) |

* Accuracy of $\pm$1 gauss.

† Number of samples.

FIG. 4.—Drawing to illustrate types of line-shapes and line-widths of signals at 35 GHz after lyophilization of tumours or muscle. Signal $S_1$ (A) is typical of muscle. Signal $S'_3$ (E) is typical of tumours. Signal $S_3$ (D) is typical of both controls and tumours after exposure to $O_2$. Signal $S_2$ (B and C) is found sporadically in both tumours and controls. Quantitative analysis was performed on the basis of $S_1$ (A) and $S'_3$ (E) type spectra. The arrows point to g = 2·004 $\pm$ 0·001.

integrated number of free radicals is similar in tumour and control samples.

The $Mn^{++}$ concentration is lower in non-lyophilized tumour samples than in muscle, but not in lyophilized samples. Because the line-shapes are all similar, quantitative measurements are based on peak–peak heights.

### *Oxygen effect on lyophilized samples*

The spectra at various times after exposure to $O_2$ (air) vary with the observing microwave frequency. At 9 GHz there is no significant change after exposure to $O_2$ for less than 5 min. After longer exposures, the signal width decreases by ~5–6 gauss for muscle (to 7·4$\pm$0·5 gauss) and 2–3 gauss for tumours (to 8·2$\pm$0·4 gauss) and remains so through the longest exposure time studied (56 h). The line-shapes are then similar for tumour and muscle samples. Fig. 5 shows spectra of samples at different exposure times. The intensities are maximal around 18 h, when the tumour samples have a peak–peak signal height about 3 × muscle. At 35 GHz, changes in line-shape are observed after about 1 min. The resulting lines are similar for both tumour and muscle ($S_3$-type signals), but the intensity of the signals is 3-fold greater for tumours than for muscle (Fig. 6).

### *Results from Experiment No 2*

In this experiment, tumours grew slowly and some eventually regressed. Histological findings are compatible with a rejection reaction. This presumably occurred because, for this experiment, we obtained Sprague-Dawley rats from a different supplier. The ESR spectra of samples from this experiment are characterized by increased amounts of NO-heme-iron triplet (Maruyama *et al.*, 1971) (Fig. 7C) and a g=2·035 NO-thio-nonheme iron protein complex (Woolum and Commoner, 1970; Vanin *et al.*, 1970) (Fig. 7A, B).

FIG. 5.—Examples of line-shape and intensity changes found at 9·1 GHz when lyophilized tumours and muscle (control) are exposed to air. All samples followed a similar pattern. In this example, the peak–peak line-width is 10 gauss for A and 13 gauss for E. The line-width narrows with exposure to ~ 8 gauss for the muscle sample and ~ 7 gauss for the tumour sample within 35–45 min, and remains approximately so for at least 50 h of exposure. Relative gain, incident microwave power and $O_2$ exposure time are indicated. Other conditions are 8 gauss modulation and 77 K.

FIG. 6.—35-GHz ESR spectra from lyophilized muscle (control) and tumour tissues exposed to $O_2$ for 17 h. The microwave power attenuation and the relative receiver gain are shown. Temperature is 123 K, modulation amplitude 8 gauss. The arrows point to g = 2·004 ± 0·001.

### DISCUSSION

These results begin to provide a basis for resolving the apparent conflicts in reports on free-radical levels in developing tumours (Swartz, 1972*a*). By studying the same samples before and after lyophilization we were able to reproduce both the apparent increase of free radicals in the early stages of tumour growth (lyophilized samples) (Emanuel, 1976; Saprin *et al.*, 1967) and a decrease in free radicals (non-lyophilized samples) (Swartz *et al.*, 1973). Our data indicate that the "increase" may be only apparent, being due to narrowing of the line-widths without change in the integrated intensities (Table II).

We did not see a late decrease in the peak–peak measurements, as reported by Saprin and co-workers (1967) and have no explanation for this discrepancy.

Our experimental method enabled us directly to compare samples before and after lyophilization, without complications from uncontrolled exposure to $O_2$ or changes in the geometry of the sample. The effects of lyophilization were found to be different for tumour and normal tissue. Comparing spectra before and after lyophilization, there were different changes in line-width (Table II), line-shape (Fig. 3) and relative integrated intensities (Table II) (*i.e.* the intensities of the spectra of non-lyophilized tumours were less than

FIG. 7.—9·1-GHz ESR spectra of 2 tumours whose histological analysis were compatible with rejection. A and B show the spectra of a 1·5-g tumour from an animal which was injected with $10^4$ cells (i.m.) 17 days previously (a 17-day tumour usually weighs 30–33 g). The peak at g = 2·03 is assigned to a non-heme–iron–sulphur–NO complex (Vanin *et al.*, 1970). Spectrum C shows a more common spectrum, due to a heme–iron–NO complex (Maruyama *et al.*, 1971). The spectrum is from 5-g tumour taken from an animal inoculated i.m. with $10^4$ cells 21 days previously. The relative gain is the same for all spectra, the microwave power is indicated. Other conditions are 8-gauss amplitude and 77 K.

those of muscle, while the intensities of the lyophilized tumour samples were the same as muscle). We conclude that (1) there is no close correlation of spectra of samples before and after lyophilization and (2) there must be physical or chemical differences in the composition of the 2 types of tissues to account for the different responses to lyophilization.

The free radicals seen after lyophilization must be at least partial artifacts because it has previously been shown that free radicals in frozen (non-lyophilized) samples closely correspond to signals in intact tissues (Ruuge *et al.*, 1976; Swartz, 1972*b*). It still may be of value to determine the molecular basis of the lyophilization signals, since their generation may be a reflection of biochemical differences between tumours and normal tissues.

The effects of $O_2$ on the spectra of the lyophilized samples were similar to those previously reported (Heckly, 1972; Ruuge *et al.*, 1976). The 35-GHz spectra of exposed samples were similar for tumour and muscle, and provide some additional detail on the nature of the radicals produced. The spectra are consistent with axially symmetrical pi-type radicals. The increased intensities after exposure to $O_2$ (Fig. 5) indicate that new radicals must be generated in the presence of $O_2$. The precursors for these radicals appear to be in high concentration in the tumour cells, because their maximum intensity was about 3× that for muscle. (Alternatively, muscle may have a higher concentration of inhibitors of the radical-generating reaction.)

Is it possible to account for the observed differences between lyophilized tumour and muscle, simply on the basis of different rates of reaction with $O_2$? This possibility is suggested by the qualitative similarity of the spectra of lyophilized tumours before $O_2$ exposure to the spectra of both tissues after each exposure (Fig. 4D, E). In that case, we must explain how, after lyophilization, there was a preferential interaction of $O_2$ with the tumour samples. Because of the high-vacuum conditions of the lyophilizations, the only source of $O_2$ could have been the samples themselves. If the outer parts of the samples were dried before all $O_2$ was removed from the centre of the sample, some $O_2$-dependent reactions could have generated radicals. There is no *a priori* reason for expecting that such an effect would have occurred preferentially in the tumours. This also would not account for the initial integrated intensity of the lyophilized tumour being the same as for muscle, nor for the concentrations of the $O_2$-dependent free radicals reaching a 3-fold higher concentration in the tumour after 18 h in air. These considerations do emphasize, however, the difficulties in attempting to interpret the results of experiments on lyophilized samples in which atmospheric $O_2$ was not rigorously excluded by lyophilizing and directly sealing samples on a high-vacuum line (Heckly, 1972). It has been shown that the usual purification procedures used to remove $O_2$ from inert gases are probably insufficient to prevent a significant $O_2$ effect in lyophilized materials (Lion & Bergmann, 1961).

We draw 2 conclusions from the data on $Mn^{++}$ summarized in Table III. Firstly, there is apparently a lower level of $Mn^{++}$ *in vivo* in the tumour. This is opposite to the changes observed in DMBA-induced mammary tumours (Swartz *et al.*, 1978: Gutierrez *et al.*, 1979). Secondly, the level of $Mn^{++}$ can be greatly affected by the processing of samples, and these effects may be different for different tissues. In our experiment, lyophilization eliminated the difference of $Mn^{++}$ concentration between muscle and tumour.

The data from the second experiment, in which the tumours grew more slowly and often regressed, confirm that both the heme–iron protein and non-heme–iron protein paramagnetic complexes with NO are more readily seen in damaged tissues. Although both these types of signal were originally proposed as specific for tumours (Brennan *et al.*, 1966; Emanuel *et al.*, 1969; Vithayathil *et al.*, 1965), they were later shown to be less specific (Woolum and Commoner, 1970; Vanin *et al.*, 1970). Occurrence of necrosis and hypoxia are important factors in their generation, such conditions being common in many tumours.

These experimental results help our understanding of the seeming contradictions in the literature on paramagnetic changes in cancer, and should make it more feasible to determine their importance. They also indictate that sophisticated spectroscopic techniques may lead to identification of the observed radical species (*e.g.* the 35-GHz data give some indications of the molecular nature of the free radicals and the strong signals seen after exposure to $O_2$ have a sufficient signal/noise ratio to make ENDOR studies feasible). But there are 2 very significant limitations to the interpretations of our data. Firstly, these results are from only one tumour system and therefore generalization is speculative. Secondly, the control tissue used in this experiment is of limited applicability. It is a common problem in studies comparing biochemical or biophysical properties of tumour and normal tissues that generally there is not a strictly comparable normal tissue. In these experiments we have used the normal tissue in to which the tumour was inoculated. The Walker tumour is poorly differentiated and has no obvious normal counterpart. The data from this experiment can be unambiguously compared only between different treatments or different times for the same tissue. Direct comparisons of tumour and muscle are speculative. Studies of other systems may reduce this problem but will not eliminate it, because by definition there cannot be identity between normal tissues and malignant tumours.

This work was supported by the National Institutes of Health Grants Nos. CA 13341 and RR 01008. Partial support was also given by the National Institutes of Health post doctoral fellowship (PLG) No. CA 05530.

### REFERENCES

ABE, H. & KURATA, Y. (1974) Spin concentration per living cell of normal liver, regenerating liver, and hepatomas. *Gann*, **65**, 75.

ANDREW, E. R. (1953) Nuclear magnetic resonance modulation correction. *Phys. Rev.*, **91**, 425.

BRENNAN, M. J., COLE, T. & SINGLEY, J. A. (1966) A unique hyperfine ESR spectrum in mouse neoplasms analyzed by computer simulation. *Proc. Soc. Exp. Biol. Med.*, **123**, 715.

BURLAKOVA, YE. B. (1967) Possible role of the free radical mechanism in regulation of cell multiplication. *Biofizika*, **12**, 91.

DIXON, W. & MASSEY, F. (1957) *Introduction to Statistical Analysis.* New York: McGraw-Hill. Chapter 9.

DUCHESNE, J., GOUTIER, R., MARECHAL, R. & VAN DEVORST, A. (1975) Free radical evolution in rat liver following the ingestion of DAB. *Phys. Med. Biol.*, **20**, 305.

EMANUEL, N. (1973) Kinetics and the free-radical mechanism of tumor growth. *Ann. N. Y. Acad. Sci.*, **222**, 1010.

EMANUEL, N. (1976) Free radicals and the action of inhibitors of radical processes under pathological states and agency in living organisms and man. *Q. Rev. Biophys.*, **9**, 283.

EMANUEL, N. M., SAPRIN, A. N., SHABALKIN, V. A., KOZLOVA, L. E. & KRUGLYKOVA, K. E. (1969) Detection and investigation of a new type of ESR signal. *Nature*, **222**, 165.

GERAU, R., GREENBERG, N., MACDONALD, M., SCHUMACHER, A. & ABBOT, B. (1972) Protocols for screening chemical agents and natural products against animal tumors and other biological systems. *Cancer Chem. Rep.*, **3**, 2.

GUTIERREZ, P., SWARTZ, H. M. & WILKINSON, E. (1979) Paramagnetic changes in cancer: a study of 7-12 dimethylbenz-α-anthracene (DMBA) induced mammary tumors using lyophilized and non-lyophilized blood, liver and tumour tissues. *Br. J. Cancer*, **39** (in press).

HALBACH, K. (1960) Modulation-effect corrections for moments of magnetic resonance line shapes. *Phys. Rev.*, **119**, 1230.

HECKLY, R. (1972) Free radicals in dry tissues. In *Biological Applications of Electron Spin Resonance*. Eds H. M. Swartz, J. R. Bolton & D. C. Borg. New York: Wiley-Interscience Inc. Ch. 5, p. 197.

KALMANSON, A. E., LIPCHINA, A. E. & CHETVERIKOV, A. G. (1961) Investigation by the method of electron paramagnetic resonance of interaction between tumor and normal cells and semiquinone ion-radicals originating from inhibitors of free-radical processes. *Biofizika*, **6**, 410.

KOLOMITSEVA, I. K., L'VOV, K. M. & KAYUSHIN, L. P. (1960) Determination of free radicals in tissues of rats inoculated with sarcoma 45. *Biofizika*, **5**, 636.

KOTRIKADZE, M., LOMSADZE, B. & TSARTSIDZE, M. (1974) Investigation of the free radical processes in the organelles of rat liver in chemical carcinogenesis. *Biofizika*, **19**, 304.

LION, M. & BERGMANN, E. (1961) The effect of oxygen on freeze-dried *Escherichia coli*. *J. Gen. Microbiol.*, **24**, 191.

MALLARD, J. & KENT, M. (1966) Electron spin resonance of surviving rat tissues. *Nature*, **210**, 588.

MARUYAMA, T., KATAOKA, N., NEGASE, S., NAKADA, N., SATO, H. & SASAKI, J. (1971) Identification of 3-Line ESR signal and its relationship to ascites tumors. *Cancer Res.*, **31**, 179.

PAVLOVA, N. L. & LIVENSON, A. R. (1965) Spectra of EPR of human blood in normal conditions and leukaemias. *Biofizika*, **10**, 169.

PETYAYEV, M. M., REZNIKOV, S. A., TERESCHENKO, T. V., CHEREPNEVA, I. YE. & SYUSINA, T. G. (1967) Kinetic investigations of the content of free radicals in lyophilized tissues of malignant tumors. *Biofizika*, **12**, 357.

RUUGE, E., KERIMOV, T. & PANEMANGLOR, A. (1976) Effect of lyophilization on the free radical states of animal cells. *Biofizika*, **21**, 124.

SAPRIN, A. N., MINEVKOV, A., NAGLER, YE. A., KAZNACHEYEV, YU. S. & EMANUEL, N. M. (1967) Course of change in the content of free radicals in developing Walker carcinosarcoma and the action of thioTEPA. *Biofizika*, **12**, 1099.

SHELLABARGER, C., CRONKITE, E., BOND, V. & LIPPINCOTT, S. (1957) The occurrence of mammary tumors in the rat after sublethal whole-body irradiation. *Radiat. Res.*, **6**, 501.

SIEGEL, S. (1956) *Nonparametric Statistics for the Behavior Sciences*. New York: McGraw-Hill. Chapt. 6.

SWARTZ, H. M. (1972*a*) Electron spin resonance studies of carcinogenesis *Adv. Cancer Res.*, **15**, 227.

SWARTZ, H. M. (1972*b*) Cells and tissues. In *Biological Applications of Electron Spin Resonance*. Eds H. M. Swartz, J. R. Bolton & D. C. Borg. New York: Wiley-Interscience Inc. Ch. 4, p. 155.

SWARTZ, H. M., ANTHOLINE, W. E. & REICHLING, B. (1978) Paramagnetic changes during development of DMBA induced mammary tumors in Sprague-Dawley rats. *Phys. Med. Biol.* (in press).

SWARTZ, H. M. & GUTIERREZ, P. (1977) Free radical increase in cancer: evidence that there is not a real increase. *Science*, **198**, 936.

SWARTZ, H. M., MAILER, C., AMBEGAONKAR, S., ANTHOLINE, W. E., MCNELLIS, D. R. & SCHNELLER, S. J. (1973) Paramagnetic changes during development of a transplanted AKR/J leukemia in mice as measured by electron spin resonance. *Cancer Res.*, **33**, 2588.

SWARTZ, H. M. & MOLENDA, R. P. (1965) Electron spin resonance spectra of some normal tissues: effect of microwave power. *Science*, **148**, 94.

TRUBY, F. & GOLDZEIHER, J. (1958) ESR investigations of rat liver and rat hepatoma. *Nature*, **182**, 1371.

VANIN, A. F., VAKHNINA, L. V. & CHETVERIKOV, A. G. (1970) Nature of the EPR signals of a new type found in cancer tissues. *Biofizika*, **15**, 1044.

VARFOLOMEYEV, V., BOGDANOV, G., D'YAKOVA, V., PAVLOVA, V. & EMANUEL, N. M. (1976) Paramagnetic properties of liver tissues and transplantable hepatomas. *Biofizika*, **21**, 881.

VITHAYATHIL, A., TERNBERG, J. L. & COMMONER, B. (1965) Changes in electron spin resonance signals of rat liver during chemical carcinogenesis. *Nature* **207**, 1246.

WALLACE, J. D. (1972) Free radicals and neoplasms, some experimental findings and some possible implications. *Crit. Rev. Biol. Sci.*, **3**, 89.

WOOLUM, J. C. & COMMONER, B. (1970) Isolation and identification of a paramagnetic complex from the livers of carcinogen treated rats. *Biochem. Biophys. Acta*, **201**, 131.